# Dipyridamole reduces penile apoptosis in a rat model of post-prostatectomy erectile dysfunction

**DOI:** 10.1590/S1677-5538.IBJU.2017.0023

**Published:** 2017

**Authors:** Omer Kutlu, Ersagun Karaguzel, Ali Ertan Okatan, Ahmet Mentese, Esin Yulug, Ilke Onur Kazaz, Selcuk Kutlu, Eyup Dil, Huseyin Eren, Ahmet Alver

**Affiliations:** 1 Department of Urology, School of Medicine, Akdeniz University, Antalya, Turkey; 2 Department of Urology School of Medicine, Karadeniz Technical University, Trabzon, Turkey; 3 Program of Medical Laboratory Techniques, Vocational School of Health Sciences. Karadeniz Technical University, Trabzon, Turkey; 4 Department of Histology and Embryology, School of Medicine, Karadeniz Technical University, Trabzon, Turkey; 5 Department of Urology, Aydin State Hospital, Aydin, Turkey; 6 Department of Biochemistry, School of Medicine, Karadeniz Technical University, Trabzon, Turkey

**Keywords:** Erectile Dysfunction, Dipyridamole, Penis

## Abstract

**Purpose:**

Despite the nerve-sparing technique, many patients suffer from erectile dysfunction after radical prostatectomy (RP) due to cavernous nerve injury. The aim of this study was to evaluate dipyridamole as a potential treatment agent of post-radical prostatectomy erectile dysfunction.

**Material and methods:**

A total of 18 male Sprague-Dawley rats were randomized into three experimental Groups (SHAM+DMSO, BCNI+DMSO and BCNI+DIP). An animal model of bilateral cavernous nerve crush injury (BCNI) was established to mimic the partial nerve damage during nerve-sparing RP. After creating of BCNI, dimethyl sulphoxide (DMSO) was administered transperitoneally as a vehicle to SHAM+DMSO and BCNI+DMSO Groups. BCNI+DIP Group received dipyiridamole (10mg/kg/day) as a solution in DMSO for 15 days. Afterwards, rats were evaluated for in vivo erectile response to cavernous nerve stimulation. Penile tissues were also analyzed biochemically for transforming growth factor-β1 (TGF-β1) level. Penile corporal apoptosis was determined by TUNEL method.

**Results:**

Erectile response was decreased in rats with BCNI and there was no significant improvement with dipyridamole treatment. TGF-β1 levels were increased in rats with BCNI and decreased with dipyridamole treatment. Dipyridamole led to reduced penile apoptosis in rats with BCNI and there was no significant difference when compared to sham operated rats.

**Conclusions:**

Although fifteen-day dipyridamole treatment has failed to improve erectile function in rats with BCNI, the decline in both TGF-β1 levels and apoptotic indices with treatment may be helpful in protecting penile morphology after cavernous nerve injury.

## INTRODUCTION

In spite of using the nerve-sparing technique, erectile dysfunction (ED) is a common complication after radical prostatectomy (RP) due to neuropraxia of the cavernous nerve (1). The main reasons which cause the cavernous nerve damage during surgery include compression, tensile and thermal damage of the nerve (2). The cavernous nerve regeneration may take several months after surgery and such a lengthy absence of innervations may lead to structural changes within the corpus cavernousum including smooth muscle loss and fibrosis (3, 4). These changes have been attributed to apoptosis after RP (5, 6). It has been well documented that smooth muscle apoptosis occurs within 24 hours after cavernous nerve injury (7). To date, some treatment approaches such as phosphodiesterase type 5 (PDE5) inhibitors, erythropoietin, FK506 (tacrolimus) have been tested for preserving the penile vascular bed and functional integrity of the cavernous nerves following RP (3, 8-10). Although they have proven some benefits for the ability to have sexual intercourse, there is still a need for new treatment approaches to prevent the penile corporeal damage mediated cavernous nerve injury.

Dipyridamole is currently used clinically as an antithrombotic drug. It increases cAMP level by inhibiting phosphodiesterase in platelet. It blocks the re-uptake of adenosine and increases the intracellular adenosine concentration (11, 12). Dipyiridamole leads to vasodilatation by increasing the adenosine formation and improves tissue perfusion in combination with antiplatelet and vasodilatory effects (13). There are also some studies that demonstrated antioxidant, neuroprotective, antiapoptotic and antifibrotic effects of dipyridamole in different tissues (14-20). Based on these features, dipyridamole may show therapeutic effect on the restoration of penile corporeal tissue after nerve-sparing RP.

In this study, an animal model of cavernous nerve crush injury was chosen to mimic the partial nerve damage during nerve-sparing RP. We aimed to evaluate the effects of daily administration of dipyridamole on penile apoptosis and erectile function measured in vivo in rats with BCNI. We also investigated the expression of transforming growth factor-β1, a well-known profibrotic cytokine that activates penile fibrosis.

## MATERIAL AND METHODS

### 

#### Animals and drugs

This experimental study was carried out in accordance with Karadeniz Technical University Animal Care and Ethic Committee directives and was approved by that committee. A total of 18 male Sprague-Dawley rats (weighing between 150 and 200g) were randomized into three experimental Groups; i) sham operation with exposure of bilateral cavernous nerves and no manipulation of the nerves plus dimethyl sulphoxide (DMSO) (SHAM+DMSO, n: 6); and ii) exposure of bilateral cavernous nerves and associated nerve injury plus DMSO (BCNI+DMSO, n: 6) and iii) exposure of bilateral cavernous nerves and associated nerve injury plus dipyiridamole (BCNI+DIP, n: 6). To create bilateral cavernous injury, animals were anesthetized with an intraperitoneal injection of a mixture of ketamine/xylazine (100+10mg/kg). The prostate was exposed via a midline abdominal incision. The cavernous nerves were identified posterolateral to the prostate. Injury was induced by applying Dumont #5 forceps (Fine Science Tools, Foster City, California, USA) to the nerve 2-3mm distal to the major pelvic ganglion. The forceps were held to closure three times for 15 seconds each, causing a moderate injury (2, 21, 22).

Following the BCNI, treatments were given according to Groups. DMSO was administered transperitoneally as a vehicle to SHAM+DMSO and BCNI+DMSO Groups for 15 days. BCNI+DIP Group received dipyiridamole (Sigma-Aldrich, St. Louis, Missouri, USA; 10mg/kg/day) as a solution in DMSO for 15 days transperitoneally. Drug dose was determined based on a report that demonstrated a protective effect of dipyiridamole in haloperidol-induced orofacial dyskinesia (23). After a 24 hours washout period, erectile responses were measured and penile tissues collected on the fifteenth day following BCNI.

#### Measurement of Erectile Responses in Vivo

After the treatment period, rats were anesthetized with a transperitoneal injection of ketamine/xylazine (100+10mg/kg) and a standard in vivo experimental protocol was conducted for evaluation of in vivo erectile response to cavernous nerve stimulation (22, 24, 25). Researchers were not aware of whether rats received placebo or dipyridamole. The carotid artery was cannulated to measure mean arterial pressure (MAP). The right penile crura was exposed and a 25G needle, connected to PE-50 tubing with 250U/mL heparin, was inserted to measure intracavernosal pressure (ICP). The cavernous nerve was identified and distal portion of nerve crushing area was stimulated with a square pulse stimulator (Grass Instruments, Quincy, Massachusetts, USA) at a frequency of 20Hz and pulse width of 50 seconds. The application of 2, 4, 6, and 8 volts was used to achieve a significant erectile response. The duration of stimulation was 1 minute with rest periods of 5 to 10 minutes between subsequent stimulations. MAP and ICP were measured with a pressure transducer (Deltran, Utah Medical Products Inc. Midvale, Utah, USA) connected to a data acquisition system (ADInstruments, Colorado Springs, California, USA). Total erectile response or total ICP was determined by the area under the erectile curve (AUC; mmHg·sec) from the beginning of cavernous nerve stimulation until the ICP pressure returned to baseline or pre-stimulation pressures. The ratio between the maximal ICP and MAP obtained at the peak of erectile response was calculated to normalize for variations in systemic blood pressure. These methods have been previously described (22, 24).

#### Measurement of Rat Transforming Growth Factor-β1 (TGF-β1) Levels

Levels of rat TGF-βΙ levels were determined by enzyme-linked immunosorbent assay kit (eBioscience-Ref No: BMS623/3, Lot No: 83382003, San Diego, California, USA), according to the manufacturer's protocols. The absorbance of samples was measured at 450nm using VERSA max tunable microplate reader (Designed by Molecular Devices, California, USA). The results were expressed as pg TGF-β1 per mL.

#### TUNEL assay

Tissue samples from the mid-shaft of the penis were harvested (n=10/group), fixed in 10% formaldehyde, embedded in paraffin, and cut in 5μm sections. Terminal deoxynucleotidyl transferase (TdT) deoxyuridine triphosphate nick end labeling assay (TUNEL) method was applied to determine the number of apoptotic cells both in smooth muscle cells and collagen fibers. TUNEL staining of sections was performed using an in situ cell death detection kit (AP kit; Roche, Mannheim, Germany), in accordance with the manufacturer's instructions. Endogenous peroxidase activity was blocked in 3% hydrogen peroxide. Color was then developed with a 3,3’-diaminobenzidine including kit (DAB, Sigma, St Louis, Missouri, USA). Two independent observers, blinded to the treatment regimen, separately evaluated apoptotic cells. Collagen fiber and muscle cells with brown nuclei were evaluated as apoptotic. TUNEL-positive cells were counted in five different fields of connective tissue and smooth muscle cells separately with 400X magnification. Quantification of TUNEL-positive cells was performed using the Analysis 5 Research program (Olympus Soft Imaging Solutions, Munster, Germany). The ratio of apoptotic nuclei to total number of nuclei was presented as the apoptotic index (AI) (26, 27).

### Statistical analysis

The statistical analyses were performed with a computer software package (GraphPad Prism^TM^ software version 5.0, La Jolla, California, USA). Data were expressed as mean±standard error of the mean (SEM). Differences between multiple Groups were compared by Kruskal-Wallis analysis of variance. Post-Hoc analyses were done by Mann-Whitney test with Bonferroni adjustment. P value of less than 0.05 was considered statistically significant.

## RESULTS

Following the BCNI, a total of 18 rats were treated with dipyridamole and placebo (DMSO) according to groups for 15 days. Mean body weights were 320.7±10.02g in SHAM+DMSO Group, 359.7±29.04g in BCNI+DMSO Group and 377.8±25.96g in BCNI+DIP Group. There were no differences in weight among the Groups at the end of the treatment (p>0.05).

**Figure 1:**
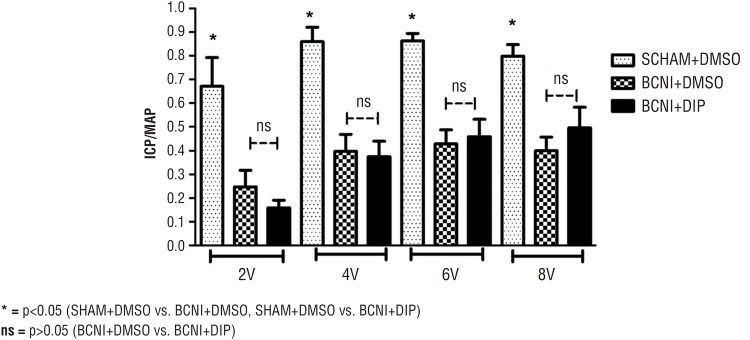
Bar graph shows voltage-dependent erectile responses as a ratio of the intracavernosal pressure (ICP) to mean arterial pressure (MAP).

There were no differences on baseline MAP and ICP levels among the Groups (respectively, p>0.05 and p>0.05). After right cavernous nerve was revealed, electrical stimulations were applied for 2, 4, 6 and 8 volts respectively to assess erection quality. Both Total ICP and ICP/MAP ratio decreased in rats with BCNI in both BCNI+DMSO and BCNI+DIP Groups as compared to sham operated rats (p <0.05). The decreased response in both total ICP and ICP/MAP of nerve injured rats were correlated for all voltages (2, 4, 6 and 8v) stimulation. After 15-day treatment with dipyridamole, there was no significant improvement in erectile response of rats in BCNI+DIP Group compared to placebo treated rats (p >0.05). Total ICP and ICP/MAP ratio of Groups are shown in Table-1 and Figure-1.

**Table 1:** Shows voltage-dependent erectile responses as measured by total ICP (area under the erectile curve [AUC; mmHg·sec]).

	SHAM+DMSO	BCNI+DMSO	BCNI+DIP	p
2v	2743±322.9	915.5±222.0	575.2±132.3	P<0.05*
4v	3318±273.8	1356±216.1	1247±196.1	P<0.05*
6v	3766±188.7	1409±202.4	1461±202.1	P<0.05*
8v	3485±191.3	1284±195.4	1606±253.2	P<0.05*

**DMSO =** dimethyl sulphoxide; **DIP =** dipyridamole; **BCNI =** bilateral cavernous nerves injury

* = p<0.05 (SHAM+DMSO vs. BCNI+DMSO, SHAM+DMSO vs. BCNI+DIP)

Penile corporal tissues of rats were evaluated biochemically in terms of TGF-β1 levels (Table-2). Fifteen days after crush injury of cavernous nerves, there was an increase in TGF-β1 levels of BCNI+DMSO Group compared to SHAM+DMSO Group (p<0.05). Dipyridamole treatment led to decrease in TGF-β1 levels of rats in BCNI+DIP Group and there was no statistical difference between BCNI+DIP Group and SHAM+DMSO Group (p>0.05).

**Table 2:** Shows the findings of TGF-β1 and penile corporal apoptosis fifteen days after bilateral cavernous nerves injury.

	SHAM+DMSO	BCNI+DMSO	BCNI+DIP	p
TGF-β1 (pg/mL)	17950±129	21490±2035	19380±432	<0.05*
Apoptotic index in smooth muscle (%)	23.50±0.80	55.33±1.68	34.17±1.47	<0.05*
Apoptotic index in connective tissue (%)	55.40±1.327	70.67±1.229	68.33±1.45	<0.05*

**TGF-β1 =** transforming growth factor-β1; **DMSO =** dimethyl sulphoxide; **DIP =** dipyridamole; **BCNI =** bilateral cavernous nerves injury.

* = SHAM+DMSO vs. BCNI+DMSO (p<0.05); SHAM+DMSO vs. BCNI+DIP (p>0.05); BCNI+DMSO vs. BCNI+DIP (p>0.05)

**Figure 2:**
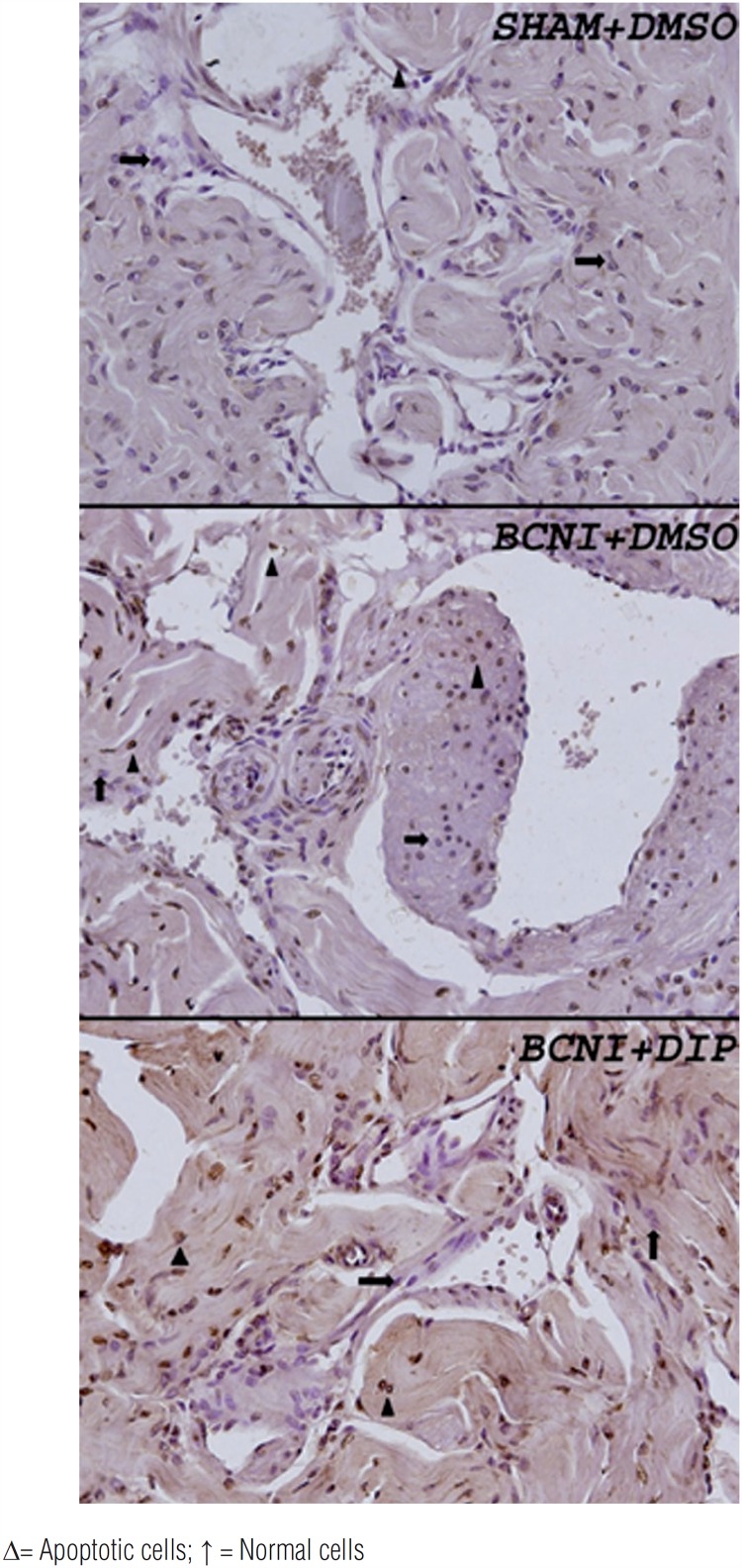
Corpus cavernousum sections stained with the TUNEL technique (original magnification 400x).

Penile apoptosis was evaluated by TUNEL method (Table-2 and Figure-2). A significant increased level of TUNEL positive cells in muscle cells and connective tissue of rats with BCNI was found compared to sham operated rats (p<0.05). The fifteen-day treatment with dipyridamole led to reduced penile apoptosis in rats with BCNI and there were no significant differences when compared to SHAM+DMSO Group (p>0.05).

## DISCUSSION

The purpose of the present study was to evaluate dipyridamole as a potential treatment agent of post-radical prostatectomy ED. A rat model of cavernous nerve crash injury was chosen to assess this hypothesis (8, 9, 28, 29).

Despite advances in nerve-sparing radical prostatectomy technique, many patients are experiencing the loss of erectile function during postoperative period (22, 30). Penile fibrosis as a result of cavernous nerve injury has been well determined in both experimental models and in patients (7, 31). Neuropraxia causes an increased apoptosis and fibrosis in both penile smooth muscle cells and endothelium (5, 6). Additionally, an increase in reactive oxygen species and TGF-β1 over expression has been reported (31-33). It has been also reported that BCNI causes a reduction in nitric oxide synthase (NOS) containing nerve fiber in animal models (34).

Even though nitric oxide (NO) is the principal relaxant of the penile smooth muscle cells, multiple other factors, either neuronal or vascular, have also been shown to modulate penile erection (35-37). Adenosine, like NO, is a potent vasodilator and its role in penile erection has been investigated in many studies which showed that intracavernous injection of adenosine resulted in tumescence and penile erection (38-41). Adenosine induced vasodilatation is mediated by increased intracellular cyclic adenosine mono phosphate (cAMP) levels in vascular smooth muscle cells via A_2_ receptor signaling (42, 43). Along with normal penile erection, adenosine signaling has also been found to be critical in erectile disorders. Gur and Ozturk suggested a greater role for adenosine as a modulator in human corpus cavernousum than in the corporal tissue of rats (44). The impairment of nonadrenergic non-cholinergic neurotransmission and endothelial dysfunction due to diabetes, chronic renal failure, and hypothyroidism, seem to contribute toward erectile dysfunction, but adenosine induced relaxation of corpus cavernousum is preserved, indicating a potential therapeutic role for adenosine (45-47). Faria et al. observed partial resistance of corpus cavernousum in men with vasculogenic impotence to adenosine induced relaxation and showed that dysfunctional A_2B_ receptors, supposedly on the endothelium, are the cause for the signaling impairment (48). Similarly, Kilic et al. observed full erection and no side effect with high dosage of intracorporeal adenosine injection in vasculogenic impotence in human (49). Chiang et al. and Filipi et al. evaluated the effect of intracorporeal injection of adenosine in impotent men which caused increased cavernosal arterial flow and resulted in suboptimal erection (50, 51). Chiang et al. attributed the suboptimal erection upon adenosine injection to the rapid degradation of adenosine by adenosine deaminase (50).

Dipyridamole is an adenosine transport inhibitor which is currently in use clinically as an antithrombotic drug. There are also some studies that demonstrated antioxidant, neuroprotective, antiapoptotic and antifibrotic effects of dipyridamole in different tissues (14-20). In a study to investigate the antioxidant properties of dipyridamole, Vargas and colleagues have found that dipyridamole probably clears the reactive oxygen radicals released from human polymorphonuclear leukocytes (ROS) (15). Also, it has been reported that dipyridamole protects the erythrocyte membranes from oxidation (52). Another animal study showed that dipyridamole protects the liver cells from the damage of ischemia/reperfusion injury (16). In addition, Garcia-Bonilla et al. identified the neuroprotective effects of dipyridamole in experimental models of cerebral ischemia in rats (17).

Dipyridamole increases cAMP level by inhibiting phosphodiesterase in platelet. It blocs the re-uptake of adenosine and increases the intracellular adenosine concentration (11, 12). Dipyiridamole leads to vasodilatation by increasing the adenosine formation and improves tissue perfusion (13). According to this knowledge, in this study, we anticipated that phosphodiesterase inhibition and adenosine accumulation by dipyridamole might contribute penile erection. But we did not see any different among the Groups after fifteen-day dipyridamole treatment. This failure might be caused by a short time for the recovery of injured cavernous nerves.

Hung et al. reported that dipyridamole has antifibrotic effect and inhibits collagen gene expression induced by TGF-β in human peritoneal mesothelial cells (20). In previous reports of animal model of BCNI, penile corporeal fibrosis has been linked to over expression of TGF-β1 (53, 54). In present study, BCNI led to increase TGF-β1 levels and fifteen-day dipyridamole treatment reduced its expression. This effect might help to reduce penile fibrosis and preserve smooth muscle cells.

Some reports have shown that dipyridamole has an antiapoptotic effect. Schrier and Yang reported that dipyridamole significantly blocks the activity of caspases which play an important role in the mechanism during the apoptotic cell death (18, 19). In our study, although we did not investigate caspase pathway, dipyridamole reduced penile corporeal apoptosis determined by TUNEL technique.

There are certainly a few limitations that are worth noting with this study. The main limitation of our study is that we have only used one time point and pharmacological dose to evaluate the therapeutic effect of dipyridamole. There is a possibility to get various outcomes with longer duration of treatment and/or different pharmacological dose. Additionally, this study did not evaluate other possible effects of dipyridamole to contribute erectile function recovery such as the antioxidant and neuroprotective effects.

## CONCLUSIONS

Although fifteen-day dipyridamole treatment has failed to improve erectile function in rats with BCNI, the decline in both TGF-β1 levels and apoptotic indices with treatment may be helpful in protecting penile morphology after cavernous nerve injury. Further studies are required to understand the effect of different pharmacological dose and long term treatment with dipyridamole especially in terms of penile hemodynamic response.
